# Association between parenteral nutrition–containing intravenous lipid emulsion and bloodstream infections in patients with single‐lumen central venous access: A secondary analysis of a randomized trial

**DOI:** 10.1002/jpen.2530

**Published:** 2023-07-10

**Authors:** Nicole C. Gavin, Emily Larsen, Naomi Runnegar, Gabor Mihala, Samantha Keogh, David McMillan, Gillian Ray‐Barruel, Claire M. Rickard

**Affiliations:** ^1^ Cancer Care Services Royal Brisbane and Women's Hospital Queensland Australia; ^2^ Alliance for Vascular Access Teaching and Research Group, School of Nursing and Midwifery Griffith University Queensland Australia; ^3^ Centre for Healthcare Transformation Queensland University of Technology Queensland Australia; ^4^ School of Nursing Queensland University of Technology Queensland Australia; ^5^ School of Nursing, Midwifery and Social Work The University of Queensland St Lucia Queensland Australia; ^6^ Faculty of Medicine University of Queensland Queensland Australia; ^7^ Nursing and Midwifery Research Centre Royal Brisbane and Women's Hospital Herston Australia; ^8^ Patient‐Centre Health Services, Menzies Health Institute Queensland Griffith University Queensland Australia; ^9^ Infection Management Services Princess Alexandra Hospital Queensland Australia; ^10^ School of Medicine and Dentistry Griffith University Queensland Australia; ^11^ School of Science, Technology, Engineering and Education; Centre for BioInnovation University of the Sunshine Coast Queensland Australia; ^12^ Herston Infectious Diseases Institute Metro North Health Herston Australia

**Keywords:** administration, intravenous, catheterization, central venous, catheter‐related infections, central venous catheters, clinical nursing research, fat emulsion, intravenous, mucosal barrier injury laboratory‐confirmed bloodstream infection, parenteral nutrition, vascular access devices

## Abstract

**Background:**

Distinguishing primary bloodstream infections (BSIs) related to central venous access devices (CVADs) from those that occur through other mechanisms, such as a damaged mucosal barrier, is difficult.

**Methods:**

Secondary analysis was conducted on data from patients with CVADs that were collected for a large, randomized trial. Patients were divided into two groups: those who received parenteral nutrition (PN)–containing intravenous lipid emulsion (ILE) and those who did not have PN‐containing ILE. This study investigated the influence of PN‐containing ILE (ILE PN) on primary BSIs in patients with a CVAD.

**Results:**

Of the 807 patients, 180 (22%) received ILE PN. Most (627/807; 73%) were recruited from the hematology and hematopoietic stem cell transplant unit, followed by surgical (90/807; 11%), trauma and burns (61/807; 8%), medical (44/807; 5%), and oncology (23/807; 3%). When primary BSI was differentiated as a central line‐associated BSI (CLABSI) or mucosal barrier injury laboratory‐confirmed BSI (MBI‐LCBI), the incidence of CLABSI was similar in the ILE PN and non–ILE PN groups (15/180 [8%] vs 57/627 [9%]; *P* = 0.88) and the incidence of MBI‐LCBI was significantly different between groups (31/180 [17%] ILE PN vs 41/627 [7%] non–ILE PN; *P* < 0.01).

**Conclusion:**

Our data indicate that twice as many primary BSIs in ILE PN patients are due to MBIs than CVADs. It is important to consider the MBI‐LCBI classification, as some CLABSI prevention efforts aimed at CVADs for the ILE PN population may be better directed to gastrointestinal tract protection interventions.

## CLINICAL RELEVANCY STATEMENT

There is a long‐standing belief that parenteral nutrition (PN) is associated with catheter‐related infections. This is the first time that the primary bloodstream infection classification of mucosal barrier injury laboratory‐confirmed bloodstream infection has been attributed to patients receiving PN‐containing intravenous lipid emulsion (ILE). Our results showed no difference between central line‐associated bloodstream infections in a group of patients who received PN‐containing ILE and those who did not have PN. This indicates that, in many patients, the microorganisms originate from the gastrointestinal tract rather than from the central venous access device.

## INTRODUCTION

Clinical guidelines for the administration of parenteral nutrition (PN)–containing intravenous lipid emulsion (ILE) (ILE PN) are more regimented than for other intravenous fluids or other component parts of PN, such as amino acids or dextrose (non–ILE PN). It is recommended that ILE PN be administered through single‐lumen central venous access devices (CVADs), or via a dedicated lumen if this is not possible, and that replacement of PN bags and associated intravenous administration sets (IVAS) occur every 12 h for solely ILE^1^, ^2^, ^3^ or every 24 h to prevent bloodstream infections (BSIs).^4^, ^5^, ^6^ There are inconsistent recommendations for IVAS replacement for non–ILE PN, ranging from every 24 h,^1^, ^2^, ^3^ every 72 h,^6^ every 96 h,^4^ or between 96 h and 168 h.^5^ Patients administered ILE PN can be considered at higher risk for BSIs, as ILEs support the growth of gram‐positive and gram‐negative bacteria and fungi,^7^ compared with other intravenous infusions, but this is only a concern if contaminated during the preparation of PN or during set up for administration.

PN‐associated BSI incidence has decreased in recent years, coinciding with improved compounding, optimal energy nutrition, and a greater understanding of glucose control,^8^ in addition to the introduction of chlorhexidine gluconate skin antisepsis and insertion bundles.^9^ Additionally, improvements in CVAD management have seen a 58% decrease in central line‐associated BSI (CLABSI) from 2001 to 2009 in the intensive care population.^10^ Similar decreases have been reported in the cancer population, with a drop in catheter‐related BSI from 56% to 25% in a 14‐year period from 1999/2000 to 2013/2014.^11^ More recently, scientific knowledge of BSI pathogenesis and definition of primary laboratory‐confirmed BSI has advanced. The Centers for Disease Control and Prevention National Healthcare Safety Network (NHSN) tracks healthcare‐associated infections in the United States, and its definitions are influential worldwide.

In 2013, NHSN introduced a new classification of laboratory‐confirmed BSI (LCBI) called mucosal barrier injury LCBI (MBI‐LCBI) to account for the translocation of gastrointestinal tract organisms in at‐risk patients, such as those with grade 3/4 gastrointestinal tract graft‐vs‐host disease undergoing allogeneic hematopoietic stem cell transplant.^12^, ^13^ The MBI‐LCBI classification is particularly relevant for patients who would otherwise be classified as having a CLABSI but are now recognized as having their primary BSI originating from their compromised mucosal barrier.^14^ Some BSIs in patients with gastrointestinal tract graft‐vs‐host disease do not result from the presence of their CVAD but, rather, from translocation of a specific list of microorganisms through nonintact mucosa. Other BSIs would be considered a secondary BSI if it has been seeded from a site‐specific infection at another body site, for example, a surgical‐site infection or pneumonia or urinary tract infection when the blood culture and primary site‐specific infection culture must have at least one matching organism within a specific time frame. With an MBI‐LCBI, there is no site‐specific infection at another body site or isolation of other common commensal organisms or recognized pathogens and the patient meets specific NHSN criteria.^14^

CVADs suspected of BSI are often removed, necessitating insertion of a new device, which further increases the risk of infective and mechanical complications. Between 70% and 85% of CVADs are likely removed unnecessarily since the resultant CVAD tip and blood cultures do not match.^15^ Diagnosing the root cause of BSI is vitally important. Distinguishing BSI related to CVADs from those occurring through other mechanisms is challenging but can facilitate BSI prevention efforts and improve reliability of benchmarking comparisons. This may also impact on our understanding of risk associated with ILE PN. The introduction of a more rigorous process for determining MBI‐LCBI will increase awareness and understanding of pathogenesis of BSI in patients receiving ILE PN and may facilitate clinical practice change. This in turn will enable more accurate BSI classification, treatment, and management in vulnerable populations, such as those diagnosed with cancer; reduce unnecessary CVAD removal and replacement; improve antimicrobial stewardship; and, consequently, reduce healthcare costs.

The aim of this study was to investigate the influence of ILE PN on catheter‐related infection in patients with CVADs. There were three objectives: (1) to compare the clinical, demographic, and treatment characteristics of patients who received ILE PN with those who did not receive ILE PN; (2) to compare infection outcomes for patients who received ILE PN and those who did not receive ILE PN; and (3) to compare microorganisms grown from blood cultures of patients who received ILE PN and those who did not receive ILE PN.

## MATERIALS AND METHODS

### Design

This is a retrospective *post hoc* cohort study using a data set from a single center involved in a multicenter randomized controlled trial.

### Setting and sample

The study included inpatients (oncology and hematology, surgical, medical, and burns and trauma) who were enrolled in *The RSVP Trial (Intravascular device administration sets: Replacement after Standard Versus Prolonged use)* ^16^, ^17^ from a quaternary teaching hospital in Queensland, Australia. Patients were followed from CVAD insertion until 48 h after device removal or discharge from the hospital with the device in situ*.*

### Inclusion/exclusion criteria

The inclusion and exclusion criteria for the parent trial are published.^16^, ^17^ Patients were excluded from the parent trial if their CVAD had been in situ for >96 h, if they had a BSI within 48 h of CVAD insertion, or if the IVAS had already been replaced. Only patients who consented to their data being used for additional analyses were included in this secondary analysis.

### Hypotheses

1. There will be significant differences in CVAD, IVAS, and patient and treatment risk‐factor data between patients who received ILE PN vs those who did not receive ILE PN.
2. There will be significantly higher rates of primary BSI outcomes (CLABSI and MBI‐LCBI) in patients who received ILE PN than in those who did not receive ILE PN.
3. There will be a difference in species of microorganisms colonizing the blood from CLABSI and MBI‐LCBI of patients receiving ILE PN compared with those who did not receive ILE PN.

### Primary outcome

Primary BSI as either a CLABSI or MBI‐LCBI.

### Primary outcome definitions

In the NHSN,^18^ a positive blood culture result satisfies the LCBI surveillance definition for a primary BSI if (1) it is determined to be healthcare‐associated, (2) it is not related to another type of healthcare‐associated infection meeting NHSN criteria (ie, not a secondary BSI), and (3) either a common commensal organism (eg, coagulase‐negative *Staphylococcus* species, viridans group *Streptococcus* species) is isolated from a blood culture on two occasions in a patient with specified signs/symptoms of BSI or a recognized pathogen (eg, *Staphylococcus aureus*, *Escherichia coli*) is isolated from at least one blood culture. If the criteria for LCBI are met and the patient has a central line in place during a specified time frame, then the LCBI is further classified as a CLABSI.

A CLABSI is a LCBI in a patient in which the central line was in place for >2 calendar days (48 h) on the date of the event, with the day of device placement being day 1.

*and*

The central line was in place on the date of the event or the day before. If the central line was in place for >2 calendar days (48 h) and then removed, the CLABSI criteria must be fully met on the day of discontinuation or the next day.

The CLABSI must meet one of the following criteria:

**Criterion 1**

The patient has a recognized pathogen cultured from one or more blood cultures.

*and*

The organism cultured from blood is **not** related to an infection at another site.

**Criterion 2**

The patient has at least one of the following signs or symptoms: fever (>38°C), chills, or hypotension.

*or*

The patient is aged younger than 1 year and has at least **one** of the following signs or symptoms: fever (>38°C core), hypothermia (<36°C core), apnoea, or bradycardia.

*and*

The organism cultured from blood is **not** related to an infection at another site.

*and*

The same (matching) potential contaminant organism is cultured from **two** or more blood cultures drawn on separate occasions.

Criterion elements must occur within a time frame that does not exceed a gap of 1 calendar day (24 h) between any two elements, eg, positive blood cultures and fever.

The same (matching) potential contaminant organisms represent a single element. The collection date of the first positive‐result blood culture should be used to determine the date of the event.

A healthcare‐associated primary BSI (ie, meeting the NHSN LCBI definition) is defined as a MBI‐LCBI if it (1) resulted from one or more of a group of selected organisms known to be commensals of the oral cavity or gastrointestinal tract and (2) occurred in a patient with certain signs or symptoms compatible with the presence of MBI. For a BSI to be classified as MBI‐LCBI, both the organism criteria and the patient criteria must be met. Eligible organisms included *Candida* species, *Enterococcus* species, *Enterobacteriaceae*, viridans group *Streptococcus* species, and certain anaerobes (*Bacteroides*, *Clostridium*, *Fusobacterium*, *Prevotella*, *Peptostreptococcus*, *Veillonella*) without isolation of additional recognized pathogens or common commensal organisms. Additionally, the BSI was required to occur in a patient with either of the following:

1. An allogeneic hematopoietic stem cell transplant in the past year and one of the following documented during the same admission as the positive blood culture result:
  a. Grades 3–4 gastrointestinal tract graft‐vs‐host disease.
  b. Diarrhea of 1 L or more in a 24‐h period documented within 7 days prior to or on the day of collection of the positive blood culture result.
2. Neutropenia meeting one of the following criteria during the 7 days prior to the collection of the positive blood culture result:
  a. Absolute neutrophil count or total white blood cell count <500/μl of blood on at least two occasions without an absolute neutrophil count of ≥500.
  b. At least one absolute neutrophil count or white blood cell count <100/μl of blood.

### Exposure of interest

The exposure of interest is ILE as defined by PN‐containing ClinOleic 20% (80% olive and 20% soya oil) (Baxter). The ILE bag and associated IVAS were replaced daily (every 24 h).

### Variables

The data extracted for this analysis included (1) administration of ILE PN during CVAD dwell; (2) CVAD data (CVAD type, number of lumens, dwell time, reason for removal); (3) IVAS data (frequency of IVAS change, number of IVAS, extension tubing); (4) patient and treatment risk‐factor data (diagnostic group, concurrent infections, blood products, immune‐suppression therapy, antineoplastic agents, intravenous medications and infusions, absolute white blood cell count, multiple organ failure, diabetes); and (5) infection outcome data classified by a blinded infectious diseases consultant (NR) after all the relevant clinical data (patient clinical observations; antimicrobial therapy; white blood cell count; CVAD tip, urine, stool, wound, or any other microscopy, culture, and sensitivity results; chest x‐ray reports; computerized tomography reports) were collated by two authors (EL and NG) and deidentified.

### Characteristics of CVAD insertion and maintenance

CVAD selection was based on medical team preference, patient treatment, patient preference, and availability of CVAD inserters. CVADs were inserted and maintained according to the standard hospital policies and procedures unless contraindicated. This included insertion under maximal sterile conditions using 2% chlorhexidine gluconate in 70% isopropyl alcohol. BIOPATCH® chlorhexidine sponges (Johnson & Johnson) were applied at the insertion or exit site under an IV3000 transparent adhesive dressing (Smith & Nephew). Peripherally inserted central catheters were secured with a StatLock® stabilization device (Bard Medical). Tunneled cuffed CVADs were sutured at the exit site, with sutures removed once the cuff had granulated. Centrally inserted central catheters were sutured. CVAD dressings were replaced weekly, unless soiled or loose, using 2% chlorhexidine gluconate in 70% isopropyl alcohol to decontaminate the skin. Totally implanted venous access devices had noncoring needles replaced weekly. If the totally implanted venous access device was not accessed, it was aspirated and flushed monthly with 0.9% sodium chloride. Needleless connectors (SmartSite™ needle‐free valve, BD) were replaced weekly or as clinically indicated. CVAD hubs (during needleless connector replacement) were decontaminated using 2% chlorhexidine gluconate in 70% isopropyl alcohol and allowed to dry. Needleless connectors and needle‐free drug ports were decontaminated with 70% isopropyl alcohol. CVADs were accessed typically daily for blood sampling (ie, complete blood cell count, biochemisty, coagulation studies. and blood transfusion grouping) with a 5–10 ml discard. All CVADs were flushed and locked with 0.9% sodium chloride using a 10 ml (or larger) syringe. A 5‐day course of 70% ethanol was prescribed to salvage CVADs in the hematology and hematopoietic stem cell transplant population when colonized with gram‐positive bacteria.

### Nutrition assessment and criteria to diagnose malnutrition

A Subjective Global Assessment was completed on admission to diagnose malnutrition. Dietetic inpatient reviews were conducted once or twice per week. Nutrition requirements and ILE PN rates for administration over a 24‐h period were calculated by the dietitian and ILE PN was prescribed by the medical team. Patients were not offered enteral nutrition alongside ILE PN. Patients were prescribed ILE PN as a single 2‐L all‐in‐one infusion, with only a minority requiring modular PN (typically for hypertriglyceridemia). In the setting of hypertriglyceridemia, a fasting triglyceride level was collected after the PN was ceased for 12 h. If the triglyceride remained high, hypertriglyceridemia was managed on an individual basis in discussion with the medical team, weighing up the risk of pancreatitis against the risk of underfeeding and deconditioning and malnutrition. Micronutrients were prescribed alongside PN. Low‐electrolyte PN formulations were prescribed for renal impairment.

Patients were generally started on their goal rate of PN (calculated to meet estimated requirements) and only started on a lower rate if they were fluid overloaded or at risk of refeeding syndrome or maintaining some oral intake. Energy and protein requirements were calculated based on 125–145 kJ/kg/day and 1.2–1.5 g protein/kg/day; adjusted body weight used if body mass index was > 25 (calculated as weight in kilograms divided by height in meters squared). The ILE rate never exceeded 110 mg/kg/h. Goal PN rate either met full requirements as estimated using above equations or supplemented oral intake to meet requirements.

### Characteristics of PN

Patients were prescribed either a single 2 L all‐in‐one PN comprising 1 L Synthamin 17 with electrolytes, 500 ml Glucose 50%, and 500 ml ClinOleic 20% (Baxter) or modular PN consisting of three separate infusions: ClinOleic 20%, Glucose 50%, and Synthamin 17 with or without electrolytes. Custom‐made PN was not available to be made in the hospital pharmacy. See Table S1 for full details.

### Characteristics of IVAS and ILE PN, non–ILE PN maintenance

#### IVAS maintenance

In keeping with the protocol of the parent study, patients were randomly assigned to either 4‐ or 7‐day IVAS replacement (burette, intravenous tubing, extension tubing, three‐way tap/stopcock, needleless connector) for maintenance and medication infusions, including non–ILE PN. IVAS for blood products and chemotherapy were discarded once the infusion was completed, in accordance with hospital procedure.

#### ILE PN and non‐ILE IVAS maintenance

The IVAS of patients requiring ILE PN were maintained by ward nurses rather than a dedicated PN team. The ILE PN bag (Baxter) and IVAS were replaced daily (every 24 h), and remaining contents were wasted at 24 h. ILE PN IVAS three‐way taps and extension tubing were left in place for either 4 or 7 days, dependent on the random allocation.

In patients with hypertriglyceridemia, the ILE PN bag and IVAS were replaced daily (every 24 h), whereas the 50% dextrose, amino acids, and associated IVAS were replaced at the randomized IVAS replacement intervals.

No in‐line filters were used, as per hospital policies and procedures. The lumen used to administer ILE PN was only accessed for blood culture sampling if the CVAD was suspected to be the source of infection; however, routine blood sampling from other lumens of the CVAD was common in cancer care.

### Pharmacy

The hospital procedure was to administer PN on a dedicated lumen, but patients who had received a hematopoietic stem cell transplant had compatible concurrent medications administered via the same IVAS. This deviation from hospital procedure is due to the complex treatment protocols for this population. In these cases, the ILE PN was connected to a lumen with compatible concurrent medications also administered, such as patient‐controlled analgesia (fentanyl, morphine, or oxycodone), potassium chloride, frusemide, or insulin, which require minimal manipulation. Each of these infusions would be administered continuously with once‐a‐day manipulation to replace the ILE PN IVAS. However, nonreturn valves were used only for patient‐controlled analgesia; therefore, ILE PN could potentially backflow into the other IVAS.

### Characteristics of care in setting of pyrexia

For patients with a temperature ≥38°C, a septic workup was undertaken. In patients with cancer, this consisted of aerobic and anaerobic blood cultures taken from a peripheral vein and each CVAD lumen, a midstream urine sample, stool sample (if indicated), CVAD site or other wound microscopy culture and sensitivities (if indicated), and a chest x‐ray. When sampling CVAD lumens for blood cultures, the initial blood draw was not discarded. Medical and surgical patients had blood cultures taken from two peripheral veins. In the hematology and hematopoietic stem cell transplant population, intravenous antibiotics were commenced within 1 h. Oncology, medical, and surgical patients had intravenous antibiotics commenced when blood culture results were reported. Research staff were not involved in the decision to take blood cultures.

### Removal of CVAD

CVADs with infective or mechanical complications were removed at the discretion of the medical team. Tunneled CVADs were removed by the medical team. Peripherally inserted central catheters and nontunneled centrally inserted central catheters were removed by ward nurses. Totally implanted venous access devices were removed surgically in the operating room. Alternatively, either the CVAD was removed at treatment completion, or the patient was discharged with the CVAD in situ for use in the outpatient or home setting.

### Sample size

The sample size was determined by patients available from the primary trial. A post hoc power calculation was performed based on observed primary BSI outcomes.

### Statistical analyses

In addition to the statistical plan outlined in the parent trial,^17^ patient data were tabulated into two groups—those who received ILE PN during their admission and those who did not—and compared using Fisher exact and Wilcoxon rank sum tests. These two groups were compared for statistically significant differences in baseline demographics, including diagnosis and immune status, as well as clinical risk data related to their IVAS and other treatment. Incidence rate ratios (IRRs) of infection were calculated to summarize the impact of ILE PN on BSI. Outcomes were compared with Kaplan‐Meier survival curves and log‐rank tests. *P* values <0.05 were considered statistically significant.

### Ethical considerations

Approval for this secondary data analysis of *The RSVP Trial* was obtained from the Human Research and Ethics Committee (HREC) of Queensland Children's Hospital (HREC/13/QRCH/185/AM13) and Griffith University (NRS/27/10/HREC).

## RESULTS

### ILE PN vs non–ILE PN patient characteristics

This secondary data analysis consisted of 807 patients with 180 (22%) receiving ILE PN (Table 1). Overall, most patients (589/807, 73%) were recruited from the hematology and hematopoietic stem cell transplant unit, followed by surgical (90/807, 11%), trauma and burns (61/807, 8%), medical (44/807, 5%), and oncology (23/807; 3%). Primary diagnoses were similar between the two groups, although patients receiving ILE PN had an absolute 10% more surgical diagnoses and 48% more allogeneic hematopoietic stem cell transplants. Risk factors for catheter‐related infection (diabetes, white blood cell count <1.0 × 10^9^/L and infection at recruitment) were balanced across the two patient groups (ILE PN and non–ILE PN).

**Table 1:** Participant and insertion characteristics by study group (*N* = 807).

	ILE received		Total	*P* value
	No	Yes		
Group size^a^	627 (78%)	180 (22%)	807 (100%)	n/c
Female sex	231 (37%)	81 (45%)	312 (39%)	0.048^b^
Age, mean (SD%)				0.003^c^
Female	53 (15%)	48 (17%)		
Male	55 (14%)	52 (17%)		
Diagnosis				<0.001^b^
Hematology	237 (38%)	12 (7%)	249 (31%)	
Autologous HSCT	101 (16%)	17 (9%)	118 (15%)	
Allogeneic HSCT	129 (21%)	93 (52%)	222 (27%)	
Surgery	56 (9%)	34 (19%)	90 (11%)	
Trauma and burns	47 (7%)	14 (8%)	61 (8%)	
Medical	37 (6%)	7 (4%)	44 (5%)	
Oncology	20 (3%)	3 (1%)	23 (3%)	
Diabetes	88 (14%)	23 (13%)	111 (14%)	0.666^b^
WBC low (<1.0 × 10^9^/L%)	63 (10%)	15 (8%)	78 (10%)	0.493^b^
Infection at recruitment	56 (9%)	9 (5%)	65 (8%)	0.088^b^
CVAD type				<0.001^d^
PICC (nt%)	398 (63%)	75 (42%)	473 (59%)	
Cuffed (t%)	214 (34%)	105 (58%)	319 (39%)	
CICC (nt%)	12 (2%)	0 (0%)	12 (2%)	
TIVAD (t%)	3 (<1%)	0 (0%)	3 (<1%)	
Number of lumens				<0.001^d^
1	17 (3%)	0 (0%)	17 (2%)	
2	485 (77%)	98 (54%)	583 (72%)	
3	123 (20%)	82 (46%)	205 (25%)	
4	2 (<1%)	0 (0%)	2 (<1%)	

*Note*: Frequencies and column percentages shown unless otherwise noted.

Abbreviations: CICC, centrally inserted central catheter; CVAD, central venous access device; HSCT, hematopoietic stem cell transplant; ILE, intravenous lipid emulsion; PICC, peripherally inserted central catheter; n/c, not calculated; nt, nontunneled; t, tunneled; TIVAD, totally implanted venous access device; WBC, white blood cell count.

^a^ Row percentages shown.

^b^ chi‐squared test.

^c^ *t* test.

^d^ Fisher exact test.

### CVAD characteristics

Peripherally inserted central catheters were the most common CVAD inserted (473/807, 59%), followed by tunneled cuffed CVADs (319/807, 39%), nontunneled centrally inserted central catheters (12/807, 2%), and totally implanted venous access devices (3/807, <1%). Patients in the ILE PN group had relatively more tunneled cuffed CVADs and fewer peripherally inserted central catheters than those in the non–ILE PN group. Most patients (583/807, 72%) had double‐lumen CVADs, with patients receiving ILE PN being approximately equally likely to have a double‐ or triple‐lumen device (Table 1). Less than 1% (8/807) of patients were admitted to the intensive care unit (Table 2). Patients who received ILE were twice as likely to be admitted to the intensive care unit (ILE PN 3/180, 2% vs non‐ILE 5/627, <1%; *P* = 0.386) (Table 2). Most patients in both groups had their CVAD removed when treatment was completed (this included patients discharged from the hospital with the CVAD in situ) with no symptoms at the CVAD exit or entry site (Table 2). The ILE PN group had a significantly longer CVAD dwell time (median 24.9 days) compared with the 17 days in the non–ILE PN group (*P* < 0.001) (Table 3).

**Table 2:** Treatment characteristics by study group (*N* = 807).

	ILE received		Total	*P* value^a^
	No	Yes		
Group size^b^	627 (78%)	180 (22%)	807 (100%)	n/c
Number of IVAS hanging and removed at the full IVAS change (total)^c^	3.0 (2.0–6.0)	11.0 (5.5–19.0)	4.0 (2.0–9.0)	<0.001
3‐way tap	487 (78%)	143 (79%)	630 (78%)	0.683
Extension tubing	451 (72%)	142 (79%)	593 (73%)	0.069
Oral antibiotics	327 (52%)	109 (61%)	436 (54%)	0.051
IV chemotherapy	342 (55%)	112 (62%)	454 (56%)	0.074
IV corticosteroids	156 (25%)	91 (51%)	247 (31%)	<0.001
IV antibiotics	484 (77%)	151 (84%)	635 (79%)	0.063
Blood products	424 (68%)	133 (74%)	557 (69%)	0.120
Nonheparinized transducer flush	8 (1%)	2 (1%)	10 (1%)	1.000
IV immune‐suppression^d^	119 (19%)	93 (52%)	212 (26%)	<0.001
Heparinized lock	0 (0%)	1 (1%)	1 (<1%)	0.223
Nonheparinized lock	161 (26%)	48 (27%)	209 (26%)	0.773
Crystalloids	621 (99%)	180 (100%)	801 (99%)	0.347
Granulocyte‐colony stimulating factor	311 (50%)	83 (46%)	394 (49%)	0.447
Prepacked medicine infusions	220 (35%)	142 (79%)	362 (45%)	<0.001
Medicine infusions (prepared on ward%)	491 (78%)	171 (95%)	662 (82%)	<0.001
Amino acids & dextrose (administered as component parts%)	3 (<1%)	26 (14%)	29 (4%)	<0.001
Propofol	4 (1%)	0 (0%)	4 (<1%)	0.580
Heparin	22 (4%)	4 (2%)	26 (3%)	0.480
Insulin infusion	2 (<1%)	4 (2%)	6 (1%)	0.025
IV bolus medications	569 (91%)	174 (97%)	743 (92%)	0.008
Admitted to ICU	5 (<1%)	3 (2%)	8 (<1%)	0.386
Therapy completed or discharged from hospital with CVAD in situ	461 (74%)	130 (72%)	591 (73%)	0.775
Number of additional VADs (peripheral and central, *N* = 515%)				0.212
0	325 (84%)	99 (79%)	424 (82%)	
1	56 (14%)	21 (17%)	77 (15%)	
2	5 (1%)	5 (4%)	10 (2%)	
≥3	3 (1%)	1 (1%)	4 (1%)	

*Note*: Percentages calculated with the number of nonmissing values in the denominator.

Abbreviations: ICU, intensive care unit; ILE, intravenous lipid emulsion; IVAS, intravenous administration set; IV, intravenous; n/c, not calculated; PN, parenteral nutrition; VAD, vascular access device.

^a^ Fisher exact test or the Wilcoxon rank sum test used.

^b^ Row percentages shown.

^c^ Median (25th–75th percentiles) shown.

^d^ Cyclosporin, tacrolimus, mycophenolate mofotil.

**Table 3:** Primary BSI outcomes by study group.

	ILE received		*P* value
	No	Yes	
Group size^a^	627 (78%)	180 (22%)	
CVAD days studied	10,948	4616	
Dwell time (CVAD days)^b^	17.0 (8.1–24.0)	24.9 (15.3–31.2)	<0.001^c^
Primary BSI^d^			
CLABSI	57 (9%)	15 (8%)	0.882^e^
IR (95% CI) per 1000 CVAD days	5.21 (4.02–6.75)	3.25 (1.96–5.39)	
IRR (95% CI)	reference	0.62 (0.33–1.12)	0.097
log‐rank test			0.011
MBI‐LCBI	41 (7%)	31 (17%)	<0.001^e^
IR (95% CI) per 1000 CVAD days	3.74 (2.76–5.09)	6.72 (4.72–9.55)	
IRR (95% CI)	reference	1.79 (1.09–2.93)	0.016
log‐rank test			0.614

*Note*: Frequencies and column percentages shown unless otherwise noted; percentages calculated with the number of nonmissing values in the denominator.

Abbreviations: BSI, bloodstream infection; CFU, colony‐forming unit; CLABSI, central line‐associated BSI; CVAD, central venous access device; ILE, intravenous lipid emulsion; IR, incidence rate; IRR, IR ratio; MBI‐LCBI, mucosal barrier injury laboratory‐confirmed bloodstream infection.

^a^ Row percentages shown.

^b^ Median (25th–75th percentiles) shown.

^c^ Wilcoxon rank sum test.

^d^ One patient was classified with a CLABSI and an MBI‐LCBI.

^e^ Fisher exact test.

### IVAS and medication characteristics

Patients in the ILE PN group had a median of 11 IVAS hanging compared with three in the non–ILE PN group. Patients in the ILE PN group received statistically significantly more intravenous corticosteroids, immune‐suppression, prepacked, medicine infusions, non–ILE PN (amino acids and dextrose), insulin infusions, and bolus medications (Table 2). Intravenous medications were prepared by registered nurses on the ward using aseptic nontouch technique, as no pharmacy containment hood was available.

### Sample size

This study of two independent samples had a post hoc power of 71% to compare the observed incidence of primary BSI with a type I error of 0.05 based on dichotomous end point.^19^

### Infection outcomes

Every patient with a positive blood culture result was classified as having a non‐LCBI, a confirmed primary BSI (CLABSI or MBI‐LCBI), a secondary BSI or a catheter‐related BSI. Positive blood culture result and microorganism type were also recorded. Only primary BSI is presented in the results. Positive blood culture result, non‐LCBI, secondary BSI and catheter‐related BSI are presented in Tables S2 and S3 and Figure S1.

#### Primary BSIs

Primary BSI cases were assigned as either a CLABSI or an MBI‐LCBI. Additional post hoc analyses were conducted presenting the infection outcomes of hematology (including autologous and heterogenous hematopoietic stem cell transplants) and nonhematology (surgery, trauma, burns, medical, and oncology) patients. The between‐group relationships were as follows:

#### CLABSI

**Figure 1:**
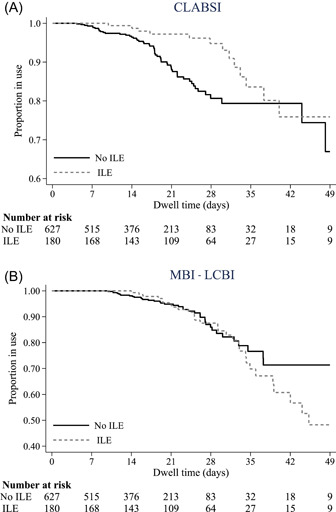
Kaplan‐Meier survival curves for central venous access device dwell time (primary bloodstream infection). CLABSI, central line‐associated bloodstream infection; ILE, intravenous lipid emulsion; MBI‐LCBI, mucosal barrier injury laboratory‐confirmed bloodstream infection.

The incidence of CLABSI was similar in the ILE PN and non–ILE PN patient groups (15/180, 8% vs 57/627, 9%; *P* = 0.882 (Table 3). Expressed as rates per 1000 CVAD days, this was 3.25 (for ILE PN) and 5.21 (for non–ILE PN) (IRR 0.62, 95% CI 0.33–1.12; *P* = 0.097 (Table 3). Kaplan‐Meier curves showed survival of CLABSI was superior in the ILE PN group between the second and fifth week (*P* = 0.011) (Figure 1A).

When the infection outcomes in hematology patients were analyzed, the incidence of CLABSI was similar to the whole data set (ILE PN 10/122, 8% vs non–ILE PN 52/467, 11%; *P* = 0.41) (Table 4). Expressed as rates per 1000 CVAD days, this was 2.82 (for ILE PN) and 5.95 (for non–ILE PN) (IRR 0.47, 95% CI 0.21–0.94; *P* = 0.022) (Table 4).

**Table 4:** Primary BSI outcomes in hematology patients by study group.

	IVFE received		*P* value
	No	Yes	
Group size^a^	467 (79%)	122 (21%)	
CVAD days studied	8728	3541	
Dwell time (CVAD days)^b^	18.2 (10.8–24.8)	27.5 (22.5–33.1)	<0.001^c^
Primary BSI^e^			
CLABSI	52 (11%)	10 (8%)	0.410^d^
IR (95% CI) per 1000 CVAD days	5.95 (4.54–7.82)	2.82 (1.52–5.25)	
IRR (95% CI)	Reference	0.47 (0.21–0.94)	0.022
Log‐rank test			0.002
MBI‐LCBI	41 (9%)	31 (25%)	<0.001^d^
IR (95% CI) per 1000 CVAD days	4.70 (3.46–6.38)	8.75 (6.16–12.4)	
IRR (95% CI)	Reference	1.86 (1.13–3.05)	0.011
Log‐rank test			0.802

*Note*: Frequencies and column percentages shown unless otherwise noted; percentages calculated with the number of nonmissing values in the denominator.

Abbreviaitons: BSI, bloodstream infection; CFU, colony‐forming unit; CLABSI, central line‐associated BSI; CVAD, central venous access device; IR, incidence rate; IRR, IR ratio; IVFE, intravenous fat emulsion; MBI‐LCBI, mucosal barrier injury laboratory‐confirmed BI.

^a^ Row percentages shown.

^b^ Median (25th–75th percentiles) shown.

^c^ Wilcoxon rank sum test.

^d^ Fisher exact test.

^e^ One patient was classified with a CLABSI and an MBI‐LCBI.

When hematology patients were removed from the analysis, the incidence of CLABSI in the nonhematology cohort was not statistically different (ILE PN 5/58, 9% vs non–ILE PN 5/160, 3%; *P* = 0.135) (Table S4), occurring at 4.65 and 2.25 per 1000 CVAD days, respectively (IRR 2.07, 95% CI 0.48–8.97; *P* = 0.268) (Table S4).

Hematology infection outcomes (not primary BSI) are presented in Table S4. Nonhematology patients were removed from the analysis, and this information is presented in Table S5.

#### MBI‐LCBI

The incidence of MBI‐LCBI was significantly different between groups (31/180, 17% for ILE PN vs 41/627, 7% for non–ILE PN; *P* < 0.001) (Table 3), occurring at 6.72 and 3.74 per 1000 CVAD days, respectively (IRR 1.79, 95% CI 1.09–2.93; *P* = 0.016) (Table 3). Survival from MBI‐LCBI was not significantly different between groups (*P* = 0.614) (Figure 1B).

There were no cases of MBI‐LCBI in the nonhematology (surgery, trauma, burns, medical, and oncology) patients.

## DISCUSSION

This secondary data analysis is the largest prospective study of PN patients within a study comparing ILE PN and non–ILE PN patients and strongly suggests that ILE PN or the frequency of IVAS replacement is not the risk; rather, it is the type of patients typically requiring long‐term intravenous therapy. Equally important, it is the first study to consider the application of the MBI‐LCBI definition in the ILE PN population and to show that CLABSI rates drop by two‐thirds with the use of this definition.

This secondary data analysis found that patients receiving ILE PN had two fewer CLABSI cases per 1000 CVAD days than non–ILE PN patients, with significantly better survival from CLABSI over time. This difference became apparent from day 7 of dwell. This contrasts with the prevailing thought that ILE PN is associated with a higher risk of CLABSI in critically ill, hospitalized, and home PN patients.^7^, ^20^, ^21^, ^22^, ^23^, ^24^, ^25^, ^26^ The results of this secondary data analysis maybe the catalyst to examine this clinical question with contemporary research and provide evidence for consistency across future clinical practice guidelines. This traditional association may have in part been driven by the inclusion of patients in CLABSI outcomes who in fact had a bacteremia due to MBI, profound neutropenia, and gastrointestinal graft‐vs‐host disease. Other explanations include improvements in PN processes in the last 1–2 decades since the studies were published, the higher proportion of tunneled CVADs in the ILE PN group, and the predominance of patients with allogeneic hematopoietic stem cell transplant within this cohort, meaning they were at greater overall risk of infection.

A large effect was seen when excluding MBI‐LCBI from the CLABSI classification, with MBI‐LCBI rates per 1000 CVAD days significantly higher in the ILE PN group. In our cohort, which comprised three‐quarters of hematology patients, including patients with allogeneic hematopoietic stem cell transplant, twice as many primary BSIs in patients receiving ILE PN were due to MBI rather than the CVAD. A post hoc analysis of infection outcomes in the nonhematology (surgery, trauma, burns, medical, and oncology) patients was conducted. This revealed a doubling of CLABSI cases per 1000 CVAD days in the patients in the non–ILE PN compared with the ILE PN group. The results of this analysis may be more representative of published studies that predated the introduction of the MBI‐LCBI classification.

It appears that microorganisms translocated from the damaged mucosal barrier may play a more important role than previously considered. Some recent studies are retrospectively reporting primary BSIs and classifying BSIs as either CLABSI or MBI‐LCBI; these studies are revealing that 44%–71% of primary BSIs in the oncology hematology and hemopoietic stem cell transplant populations are MBI‐LCBI^27^, ^28^, ^29^, ^30^, ^31^, ^32^, ^33^ and between 8% and 10.5% in the general hospital population.^28^, ^34^

The characteristics of patients who received ILE PN compared with those who did not receive ILE PN were also revealing and explain the significantly higher incidence of MBI‐LCBI in patients receiving ILE PN. The difference in median dwell time was statistically significant between groups, with ILE PN patients having their CVADs in situ approximately 8 days longer than their non–ILE PN counterparts. Patients in the ILE PN group had almost four times the number of IVAS, which indicated their higher acuity. About half (93/180, 52%) of all ILE PN patients received an allogeneic hematopoietic stem cell transplant compared with 21% (129/627) of non–ILE PN patients. This group of patients were prescribed ILE PN when they had severe mucositis and esophagitis and were unable to tolerate oral feeding. This summary of patient characteristics demonstrates that patients who received ILE PN have a worse nutrition status and were more acutely unwell and immunocompromised, necessitating longer CVAD dwell.

Despite this, the percentage of CVAD removal for suspected BSI and diagnosed catheter‐related infection outcomes was not worse in the ILE PN group. Patients receiving ILE PN were prone to more positive‐result blood culture of *Candida* species and gram‐positive bacilli. Gavin and colleagues^35^ analyzed the pathogens isolated from blood cultures of patients who received PN and those who did not. Patients receiving ILE PN were more likely to have fungal or polymicrobial microorganisms and less likely to have gram‐positive cocci compared with patients receiving no‐ILE PN. In our secondary data analysis, the distribution of microorganisms between the two groups was less marked, as the only fungal infections, including yeasts and molds, (*n* = 3) were present in the ILE PN group. The predominance of hematology patients with indications for antifungal prophylaxis may have contributed to this finding in our cohort.

Our study is the first to consider MBI‐LCBI in relation to CVADs and PN administration. Other studies^14^, ^27^, ^28^, ^31^ of primary BSI in other populations that included the MBI‐LCBI definition have similarly reported that CLABSI rates have halved when attributing the MBI‐LCBI classification. None of these studies reported patient characteristics with regard to nutrition. The incidence of CLABSI is expected to drop in coming years when more appropriate definitions are applied. In the interim period, when not all institutions have applied the MBI‐LCBI classification, researchers will need to be cognizant of this when meta‐analyzing data from published studies. Researchers should describe the clinical characteristics of the patient groups and clinical care to enable reliable meta‐analysis and subgroup analyses in future systematic reviews.

Gavin and colleagues^35^ undertook a systematic review of comparative rates of catheter‐related BSI in patients with CVADs who received ILE PN and those who did not receive ILE PN. This systematic review^35^ comprised 11 studies, of which only one analyzed catheter‐related BSI per patient.^36^ Penel and colleagues^36^ recruited 371 patients with a small proportion receiving ILE PN (29/371, 8.5%). Patients receiving ILE PN were more likely to develop a catheter‐related BSI (odds ratio 10.9, 95% CI 3.48–34.05). Four studies^36^, ^37^, ^38^, ^39^ included in this systematic review recruited 911/2854 (32%) oncology and 195/2854 (7%) hematology patients, but because of reporting, it was not possible to compare catheter‐related BSIs between diagnostic groups. Since this systematic review was published, two further retrospective studies have reported CLABSIs in patients receiving ILE PN and no‐ILE PN^40^, ^41^ with 122/4839 (2.5%) and 767/38,674 (<2%) receiving ILE PN with an odds ratio of 4.33 and 2.65, respectively. The inclusion of these two studies in the systematic review would provide further corroborating and significant results in favor of non–ILE PN for CLABSI. None of these studies differentiated between CLABSI and MBI‐LCBI. In our secondary data analysis, we observed that patients who had received ILE PN were less likely to develop a CLABSI compared with the patients who had never received ILE PN during the CVAD dwell time (OR 0.91, 95% CI 0.5–1.65). All studies reported above occurred before the introduction of the MBI‐LCBI classification; therefore, comparisons between their results and the secondary data analysis are not justified.

This secondary data analysis was planned, and data were collected prospectively, in tandem with the parent randomized controlled trial, which diminishes the opportunity for observer bias. A limitation of this study is that it is a secondary data analysis from one hospital that was involved in a multisite randomized controlled trial. Furthermore, information about some confounders, such as routine blood sampling and blood cultures or nutrition status, was not collected as part of the randomized controlled trial. Group assignment to ILE PN or non–ILE PN was observed, not manipulated, which is an acknowledged limitation of the observational design. These results need to be interpreted with caution and do not provide evidence of cause and effect, as secondary data analyses are nonrandomized comparisons and patient and/or clinical characteristics may influence the results. Therefore, they cannot provide a definitive link between risk factors and health outcomes. Additionally, a multivariate Cox regression analysis was attempted, but many variables were correlated, had interaction effects, or did not meet the proportional hazards assumption; therefore, the analysis could not be performed. Thus, the significance of CLABSI in the ILE PN group regarding covariates and imbalances could not be determined. There were also several significant baseline differences between the ILE PN and non–ILE PN groups.

This secondary data analysis is the next step in understanding the long‐standing clinical question about the infection risk attributable to ILE PN. Future research needs to focus on the translocation of microorganisms from the gastrointestinal tract to the bloodstream and, in turn, into the CVAD. Researchers should collect the additional confounders highlighted above, as well as more detailed diagnostic information, as patients with a bowel obstruction are also at risk of translocation. As the microbiome of the gastrointestinal tract differs to that on the skin, the identification at species level would be a strong indicator. To date, researchers have studied the changes in bacterial communities in the gastrointestinal tract of patients undergoing induction chemotherapy for acute myeloid leukemia^42^ and their allogeneic hematopoietic stem cell transplant.^43^, ^44^ It is important that future research includes fungal, as a minimum, bacterial, as standard, and viral, if possible, communities. Hematology and hematopoietic stem cell transplant patients are prescribed prophylactic antibiotics, antifungals, antivirals, and proton pump inhibitors, which impact on the gastrointestinal tract microbiome. Understanding fungal as well as bacterial communities would facilitate greater understanding of hematogenous seeding in catheter‐related infection pathogenesis. This shift in the focus of research and treatment would, in turn, improve CLABSI prevention strategies, as there would be a greater understanding of the significance of the translocation of gastrointestinal tract microorganisms in this population.

This study set out to answer three objectives: (1) to compare the clinical, demographic, and treatment characteristics of patients who received ILE PN with those who did not receive ILE PN; (2) to compare infection outcomes for patients who received ILE PN and those who did not receive ILE PN; and (3) to compare microorganisms grown from blood cultures of patients who received ILE PN and those who did not receive ILE PN.

### CONCLUSION

Patients who receive ILE PN have a worse nutrition status and are more acutely unwell and immunocompromised, necessitating longer CVAD dwell; ILE PN was associated with decreased CLABSI in this single‐center secondary data analysis once MBI‐LCBI diagnoses were applied and time at risk was considered; and patients receiving ILE PN were prone to more positive‐result blood cultures of *Candida* species and gram‐positive bacilli. It is time to reexamine the safety of extending the replacement of IVAS for ILE PN beyond 24 h. This clinical question needs to be examined with a randomized controlled trial to provide level II evidence and inform future systematic reviews and meta‐analyses. Striving for standardization in clinical guidelines and practice for IVAS across all intravenous solutions is the goal.

## AUTHOR CONTRIBUTIONS

Nicole Gavin, Samantha Keogh, David McMillan, and Claire Rickard contributed to conception/design of the research; Nicole Gavin, Emily Larsen, Naomi Runnegar, Gabor Mihala, Samantha Keogh, David McMillan, Gillian Ray‐Barruel, and Claire Rickard contributed to acquisition, analysis, or interpretation of the data; Nicole Gavin drafted the manuscript; and Nicole Gavin, Emily Larsen, Naomi Runnegar, Gabor Mihala, Samantha Keogh, David McMillan, Gillian Ray‐Barruel, and Claire Rickard critically revised the manuscript. All authors agree to be fully accountable for ensuring the integrity and accuracy of the work. All authors read and approved the final manuscript.

## CONFLICT OF INTEREST STATEMENT

Nicole C. Gavin received educational funding and honoraria from ICU Medical and Hospira. Emily N. Larsen's employer has received, on her behalf, an investigator‐initiated research grant from Cardinal Health (formerly Medtronic) and Eloquest Healthcare and an educational (conference) scholarship from Angiodynamics. Gillian Ray‐Barruel reports speaker fees provided to Griffith University by product manufacturers (3M, Becton Dickinson) and education providers (Ausmed, Wolters Kluwer, Continulus), unrelated to this project. Claire M. Rickard reports investigator‐initiated grants and consultancy payments made to her employer (Griffith University of University of Queensland) from 3M, BBraun, BD‐Bard, Cardinal Health, Eloquest, and ITL Biomedical.

## Supporting information

Figure S1: Kaplan‐Meier survival curves for CVAD dwell time (CRBSI).

Supporting information.
