# Supplementary figures and images for: Association between parenteral nutrition–containing intravenous lipid emulsion and bloodstream infections in patients with single‐lumen central venous access: A secondary analysis of a randomized trial
Source: JPEN J Parenter Enteral Nutr. 2023 Jul 10;47(6):783–95. doi: 10.1002/jpen.2530 (PMC10946626; doi:10.1002/jpen.2530)

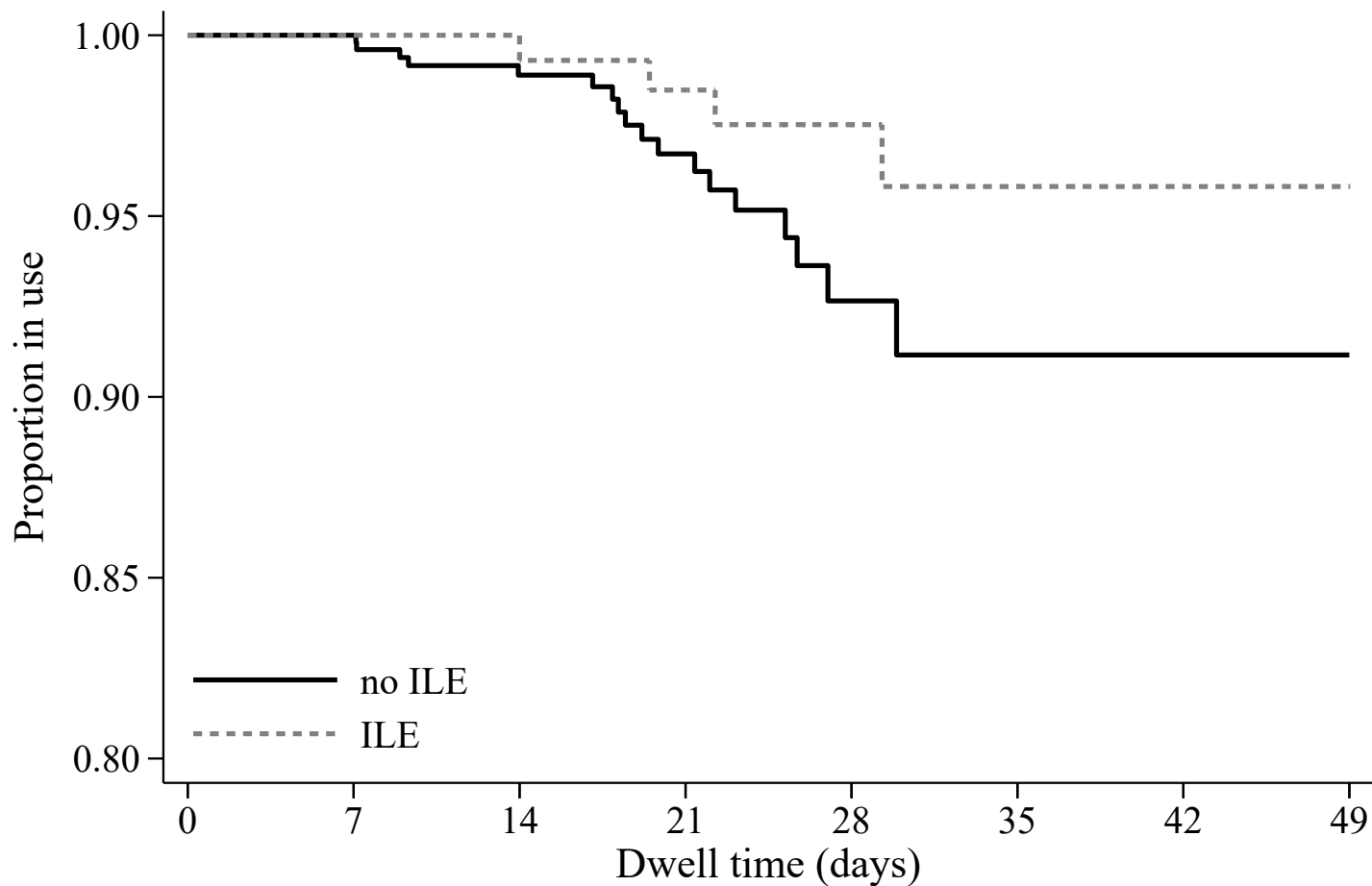

**Number at risk**

|        |     |     |     |     |    |    |    |   |
|--------|-----|-----|-----|-----|----|----|----|---|
| no ILE | 627 | 515 | 376 | 213 | 83 | 32 | 18 | 9 |
| ILE    | 180 | 168 | 143 | 109 | 64 | 27 | 15 | 9 |

Supplement: Supplementary file 1 — Figure S1: Kaplan‐Meier survival curves for CVAD dwell time (CRBSI). [file JPEN-47-783-s001.pdf]
