# Supplementary material for: Association between parenteral nutrition–containing intravenous lipid emulsion and bloodstream infections in patients with single‐lumen central venous access: A secondary analysis of a randomized trial
Source: JPEN J Parenter Enteral Nutr. 2023 Jul 10;47(6):783–95. doi: 10.1002/jpen.2530 (PMC10946626; doi:10.1002/jpen.2530)
Supplement: Supplementary file 2 — Supporting information. [file JPEN-47-783-s002.docx]

#### Supplementary Table S1: Characteristics of PN

|  | PN (all-in-one) | Clinoleic 20% | Synthamin 17 (amino acid 10%) with electrolytes | Synthamin 17 (amino acid 10%) without electrolytes | Glucose 50% |
| --- | --- | --- | --- | --- | --- |
| Volume | 2L | 500mL | 500mL | 500mL | 500mL |
| Components | 1l Synthamin 17 with electrolytes  500ml Glucose 50%  500ml Clinoleic 20% | 20% triglyceride  1.2% phospholipid  2.25% glycerol | N/A | N/A | 250g anhydrous glucose  Water for injection |
| Total nutrients | 100g amino acids (16.5g nitrogen)  100g lipid  250g glucose | N/A | N/A | N/A | N/A |
| Total electrolytes | 73mmol sodium  60mmol potassium  5mmol magnesium  3.75mmol calcium  70mmol chloride  37.5mmol phosphate  150mmol acetate | N/A | N/A | N/A | N/A |
| Total energy | 9860Kj  1700Kj nitrogen  4180Kj lipid  3980Kj carbohydrate | N/A | N/A | N/A | N/A |
| Active ingredients | N/A | 100g 80:20% refined olive oil & soya oil | N/A | N/A | N/A |
| Excipients |  | 11.25g glycerol  6g egg lecithin  3mg ascorbyl palmitate  150mg sodium oleate  Sodium hydroxide qs pH 6-8  500ml water for injection | N/A | N/A | N/A |
| Essential amino acids | N/A | N/A | 3.65g L-leucine  3g L-isoleucin  2.9g L-lysine (added a hydrochloride salt)  2.9g L-valine  2.8g L-phenylalanine  2.4g L-histidine  2.1g L-threonine  2g L-methionine  0.9g L-tryptophan | 3.65g L-leucine  3g L-isoleucin  2.9g L-lysine (added a hydrochloride salt)  2.9g L-valine  2.8g L-phenylalanine  2.4g L-histidine  2.1g L-threonine  2g L-methionine  0.9g L-tryptophan | N/A |
| Non-essential amino acids | N/A | N/A | 10.35g L-alanine  5.75g L-arginine  5.15g Glycine  3.4g L-proline  2.5g L-serine  0.2g L-tyrosine | 10.35g L-alanine  5.75g L-arginine  5.15g Glycine  3.4g L-proline  2.5g L-serine  0.2g L-tyrosine | N/A |
| Electrolytes | N/A | N/A | 3.4g sodium acetate  2.61g dibasic potassium phosphate  0.585g sodium chloride  0.51g magnesium chloride  Water for injection QS  36.5mmol sodium  30mmol potassium  2.5mmol magnesium  75mmol acetate  35mmol chloride  15mmol phosphate  8.25g/500ml nitrogen content | N/A | N/A |
| pH | Not stated | 6-8 – isotonic emulsion | 5.5-6.5 | 5.5-6.5 | 3.2-6.5 |
| Osmolarity | Not stated | 345 mOsm/kg | 1260 mOsm/kg | Not stated | 1000 mOsm/kg |

Table S2: Infection outcomes (not including primary BSI) by study group

|  | **ILE received** | | ***p*-value** |
| --- | --- | --- | --- |
|  | **no** | **yes** |  |
| Group size^a^ | 627 (78%) | 180 (22%) |  |
| CVAD-days studied | 10,948 | 4,616 |  |
| Dwell time (CVAD-days)^b^ | 17.0 (8.1–24.0) | 24.9 (15.3–31.2) | <0.001^c^ |
| Positive blood culture (of any kind) | 176 (28%) | 76 (42%) | <0.001^d^ |
| Non-LCBI^e^ | 68 (11%) | 27 (15%) | 0.149^d^ |
| Secondary BSI | 11 (2%) | 3 (2%) | 1.000^d^ |
| CRBSI | 18 (3%) | 4 (2%) | 0.798^d^ |
| IR (95% CI) per 1,000 CVAD-days | 1.64 (1.04–2.61) | 0.87 (0.33–2.31) |  |
| IRR (95% CI) | reference | 0.53 (0.13–1.60) | 0.246 |
| log-rank test |  |  | 0.119 |
| CVAD tip cultured by pathology (*N*=92): |  |  |  |
| tip culture growth >15 CFU | 5 (<1%) | 3 (2%) | 0.698^d^ |
| tip culture growth ≤15 CFU | 5 (1%) | 2 (1%) | 1.000^d^ |
| tip no growth | 59 (9%) | 18 (10%) | 0.178^d^ |
| *Note: frequencies and column percentages shown unless otherwise noted; percentages calculated with the number of non-missing values in the denominator; ^a^ row percentages shown; ^b^ median (25th–75th percentiles) shown; ^c^ Wilcoxon rank-sum test; ^d^ Fisher’s exact test; ^e^ 14 patients had more than one common contaminant; BSI = bloodstream infection; CFU = colony-forming unit; CI = confidence interval; CRBSI = catheter-related BSI; CVAD = central venous access device; IR = incidence rate; IRR = incidence rate ratio; ~~IVFE~~ ILE = intravenous ~~fat~~ lipid emulsion; LCBI = laboratory confirmed bloodstream infection.* | | | |

Supplementary Table S2: Individual patient positive blood culture results tabulated into common commensal, primary BSI, secondary BSI, CRBSI and CVAD tip

|  | No-ILE | ILE |
| --- | --- | --- |
| **non-LCBI:** |  |  |
| central | Corynebacterium sp. (338†)  Dermabacter hominis (602)  Microbacterium sp. (408)  Moraxella catarrhalis (354)  Staphylococcus capitis (629†)  Staphylococcus epidermidis (284; 291; 296; 334†; 389; 421; 449; 479; 573; 581; 610; 625; 629†; 648; 651; 653; 684; 688; 707; 739; 1061; 1064; 1125; 1137; 1164†; 1248)  Staphylococcus haemolyticus (351; 494; 500; 577; 584; 665; 668; 686; 1011; 1028†; 1054; 1164†)  Staphylococcus hominis (292; 358; 432; 556†; 595; 1013; 1085†)  Staphylococcus warneri (456; 654)  Coag. neg. Staphylococcus (650; 675; 677)  Mixed coagulase neg. Staphylococcus sp. (376)  Streptococcus gordonii (1085†)  Streptococcus salivarius (1308)  Streptococcus sp. (alpha-haemolytic) (420†)  Micrococcus sp.; Staphylococcus capitis (556†)  Staphylococcus capitis; Staphylococcus simulans (384)  Staphylococcus epidermidis; Staphylococcus haemolyticus (467)  Staphylococcus epidermidis; Staphylococcus haemolyticus; Staphylococcus hominis; (473)  Staphylococcus haemolyticus; Staphylococcus hominis (1224) | Bacillus cereus (678†; 1065†; 1084)  Corynebacterium sp. (1059; 1108)  Staphylococcus capitis (258; 275; 1313†)  Staphylococcus cohnii (1195)  Staphylococcus epidermidis (355; 386; 544†; 619; 692†; 1006; 1080; 1302)  Staphylococcus haemolyticus (230†; 304; 692†)  Staphylococcus hominis (678†; 1065†; 1188; 1313†)  Coag. neg. Staphylococcus (285; 1200)  Sphingomonas paucimobilis (545)  Corynebacterium sp.; Staphylococcus haemolyticus (545) |
| peripheral | Staphylococcus capitis (338†)  Staphylococcus epidermidis (420†)  Staphylococcus haemolyticus (1273)  Coag. neg. Staphylococcus (1183)  Mixed coag. neg. Staphylococcus sp. (334†) | Micrococcus sp. (604)  Staphylococcus capitis (1270†)  Staphylococcus warneri (230†)  Mixed coag. neg. Staphylococcus (1270†)  Streptococcus mitis (732) |
| not documented | Bacillus sp. (1028†)  Corynebacterium jeikeium (1144)  Staphylococcus epidermidis (1150)  Staphylococcus haemolyticus (618; 1319)  Staphylococcus warneri (1109)  Coag. neg. Staphylococcus (1087; 1294) |  |
| **Primary BSI** |  |  |
| CLABSI | Acinetobacter baumannii complex (281)  Acinetobacter sp. (667)  Alloiococcus otitidis (1135)  Enterobacter cloacae (416; 541; 1143)  Escherichia coli (639; 1053)  Klebsiella oxytoca (434; 599)  Klebsiella pneumoniae (431)  Pseudomonas aeruginosa (184; 1112; 1241)  Serratia marcescens (649)  Staphylococcus aureus (1025)  Staphylococcus epidermidis (446; 528; 606; 652)  Staphylococcus haemolyticus (212; 611; 1269)  Stenotrophomonas maltophilia (548)  Streptococcus mitis (455)  Acinetobacter baumannii complex; Staphylococcus haemolyticus (563)  Citrobacter koseri; Pseudomonas aeruginosa (267)  Clostridium sp.; Staphylococcus epidermidis; Streptococcus mitis (499)  Corynebacterium sp.; Staphylococcus aureus (1203)  Enterobacter cloacae; Ps. (Sphingomonas) paucimobilis; Coag. neg. Staphylococcus (1297)  Enterobacter cloacae; Staphylococcus epidermidis; Staphylococcus haemolyticus (427)  Enterococcus faecalis; Pseudomonas aeruginosa (413)  Enterococcus faecalis; Staphylococcus aureus; Streptococcus mitis (630)  Escherichia coli; Pseudomonas aeruginosa (544†)  Gemella sp.; Staphylococcus haemolyticus (720)  Granulicatella (Abiotrophia) adiacens; Streptococcus mitis (1190)  Klebsiella oxytoca; Serratia marcescens; Staphylococcus epidermidis; Stenotrophomonas maltophilia (533)  Pseudomonas aeruginosa; Staphylococcus aureus (703)  Pseudomonas aeruginosa; Staphylococcus capitis (380)  Pseudomonas putida; Staphylococcus epidermidis (689)  Pseudomonas aeruginosa; Streptococcus mitis (1255)  Staphylococcus aureus; Staphylococcus epidermidis (299)  Staphylococcus aureus (MRSA); Streptococcus mitis (447)  Staphylococcus capitis; Pseudomonas aeruginosa; Pseudomonas aeruginosa (1168)  Staphylococcus capitis; Staphylococcus hominis (568)  Staphylococcus epidermidis; Staphylococcus haemolyticus (671; 1267)  Staphylococcus epidermidis; Staphylococcus haemolyticus; Pseudomonas putida (725)  Staphylococcus epidermidis; Stenotrophomonas maltophilia (1191)  Staphylococcus epidermidis, Staphylococcus hominis (1038)  Staphylococcus epidermidis;  Staphylococcus warneri (616)  Staphylococcus epidermidis; Streptococcus agalactiae (Group B) (307)  Staphylococcus haemolyticus; Staphylococcus hominis (633; 638)  Staphylococcus lugdunensis; Staphylococcus haemolyticus; Staphylococcus hominis; Streptococcus agalactiae (Group B) (710)  Coagulase Neg. Staphylococcus; Stenotophamonas maltophilia (1299)  Streptococcus pneumoniae; Streptococcus viridians (1208) | Enterobacter cloacae (708)  Pseudomonas (Sphingomonas) paucimobilis (1031)  Staphylococcus aureus (1161)  Staphylococcus epidermidis (1202; 1312)  Staphylococcus haemolyticus (403; 637; 1111)  Staphylococcus warneri (190)  Acinetobacter sp.; Escherichia coli;  Staphylococcus capitis (1118)  Bacteroides fragilis; Enterococcus faecium; Escherichia coli (551)  Candida glabrata complex; Klebsiella oxytoca (1304)  Corynebacterium sp.; Mixed coag. neg. Staphylococcus sp. (385)  Escherichia coli; Klebsiella pneumoniae; Staphylococcus hominis (391)  Klebsiella oxytoca; Staphylococcus haemolyticus (1206) |
| MBI-LCBI | Citrobacter freundii (657)  Clostridium septicum (298; 520)  Enterobacter cloacae (177; 492; 679)  Enterococcus faecium (462; 1262)  Enterococcus faecium (VRE) (333; 1289)  Escherichia coli (417; 439; 443; 486; 1145; 1301)  Escherichia coli (ESBL) (480; 1307)  Klebsiella pneumoniae (419; 1068)  Pseudomonas aeruginosa (469; 524)  Streptococcus salivarius (238)  Streptococcus mitis (372; 470; 1124; 1184)  Capnocytophaga sputigena; Fusobacterium nucleatum (1285)  Enterobacter cloacae; Enterococcus faecium (VRE); Fungus sp.; Klebsiella pneumoniae; Pseudomonas aeruginosa (395)  Enterococcus faecium (579)  Escherichia coli (1316)  Klebsiella oxytoca; Enterobacter cloacae (534)  Klebsiella pneumoniae; (1218)  Klebsiella pneumoniae (410)  Klebsiella pneumoniae (ESBL) (512)  Klebsiella pneumoniae; Streptococcus mitis (641)  Pseudomonas aeruginosa; Pantoea sp. (1166)  Serratia marcescens (451)  Streptococcus mitis (561; 1221)  Streptococcus salivarius (696) | Candida glabrata complex (1042)  Candida krusei (yeast) (1194)  Enterococcus faecium (489; 588)  Enterococcus faecium (VRE) (1279)  Escherichia coli (1233)  Fusobacterium nucleatum (482)  Klebsiella pneumoniae (728)  Streptococcus mitis (608; 1121; 1234)  Streptococcus salivarius (518)  Capnocytophaga sp. (418)  Enterobacter cloacae; Enterobacter faecalis; Bacillus sp. (392)  Enterobacter cloacae; Escherichia coli (1222)  Enterococcus faecalis; Escherichia coli (MRO) (1072)  Enterococcus faecalis (273)  Enterococcus faecium (VRE);  Escherichia coli (1122)  Enterococcus faecium (VRE); Corynebacterium jeikeium (1056)  Enterococcus faecium (329)  Klebsiella pneumoniae; Aeromonas hydrophilia (1281)  Klebsiella pneumoniae; (1088)  Streptococcus intermedius (S. milleri gp) (585)  Streptococcus mitis (MRSA) (575)  Streptococcus mitis; (1116)  Streptococcus mitis (468; 498)  Streptococcus mitis Pseudomonas putida (436)  Streptococcus mitis; (1094)  Streptococcus mitis (1266) |
| **Secondary BSI** | Bacteroides sp. (186)  Enterobacter cloacae (450)  Enterococcus faecalis (656)  Enterococcus faecium (1012)  Escherichia coli (719)  Klebsiella pneumoniae (ESBL) (373)  Pseudomonas aeruginosa (328)  Pseudomonas aeruginosa; Staphylococcus haemolyticus (1083)  Staphylococcus aureus; Staphylococcus haemolyticus; Staphylococcus hominis (1193)  Staphylococcus aureus; Staphylococcus hominis (1055) | Bacteroides thetaiotaomicron (1131)  Escherichia coli (ESBL) (555)  Ralstonia (Burk.) pickettii; Ralstonia sp. (1082) |
| **CVAD tip** |  |  |
| growth <15 CFU | Staphylococcus epidermidis (432)  Staphylococcus hominis (420)  Coag. neg. Staphylococcus (595; 1301; 1083) | Coag. neg. Staphylococcus (708)  Mixed Skin Flora (1304) |
| growth ≥15 CFU | Acinetobacter baumannii complex (*281; *563)  Mixed skin flora (380)  Pseudomonas aeruginosa (*413; *328) | Staphylococcus aureus (MRSA) (*575)  Staphylococcus epidermidis (*1202)  Mixed coag. neg. Staphylococcus (392) |
| ** matching CVAD tip and BC; † patient had multiple common contaminants; coag. neg. = coagulase negative;* | | |

Supplementary Table S4. Infection outcomes (not primary BSI) in hematology patients by study group

|  | **ILE received** | | ***p*-value** |
| --- | --- | --- | --- |
|  | **no** | **yes** |  |
| Group size^a^ | 467 (79%) | 122 (21%) |  |
| CVAD-days studied | 8,728 | 3,541 |  |
| Dwell time (CVAD-days)^b^ | 18.2 (10.8–24.8) | 27.5 (22.5–33.1) | <0.001^c^ |
| Positive blood culture (of any kind) | 157 (34%) | 67 (55%) | <0.001^d^ |
| Non-LCBI^e^ | 59 (13%) | 24 (20%) | 0.057^d^ |
| Secondary BSI | 6 (1%) | 2 (2%) | 0.673^d^ |
| CRBSI | 15 (3%) | 4 (3%) | 1.000^d^ |
| IR (95% CI) per 1,000 CVAD-days | 1.72 (1.04–2.85) | 1.13 (0.42–3.01) |  |
| IRR (95% CI) | reference | 0.66 (0.16–2.06) | 0.477 |
| log-rank test |  |  | 0.177 |
| *Note: frequencies and column percentages shown unless otherwise noted; percentages calculated with the number of non-missing values in the denominator; ^a^ row percentages shown; ^b^ median (25^th^–75^th^ percentiles) shown; ^c^ Wilcoxon rank-sum test; ^d^ Fisher’s exact test; ^e^ 14 patients had more than one common contaminant; ^f^ one patient was classified with a CLABSI and a MBI-LCBI; BSI = bloodstream infection; CFU = colony-forming unit; CI = confidence interval; CLABSI = central line-associated BSI; CRBSI = catheter-related BSI; CVAD = central venous access device; IR = incidence rate; IRR = incidence rate ratio; IVFE = intravenous fat emulsion; LCBI = laboratory confirmed bloodstream infection; MBI-LCBI = mucosal barrier injury LCBI.* | | | |

Supplementary Table S5. Infection outcomes in non-hematology patients by study group

|  | **ILE received** | | ***p*-value** |
| --- | --- | --- | --- |
|  | **no** | **yes** |  |
| Group size^a^ | 160 (73%) | 58 (27%) |  |
| CVAD-days studied | 2,220 | 1,075 |  |
| Dwell time (CVAD-days)^b^ | 8.8 (6.0–18.4) | 13.8 (8.9–20.1) | 0.032^c^ |
| Positive blood culture (of any kind) | 19 (12%) | 9 (16%) | 0.496^d^ |
| Non-LCBI^e^ | 9 (6%) | 3 (5%) | 1.000^d^ |
| Primary BSI^f^: |  |  |  |
| CLABSI | 5 (3%) | 5 (9%) | 0.135^d^ |
| IR (95% CI) per 1,000 CVAD-days | 2.25 (0.94–5.41) | 4.65 (1.94–11.2) |  |
| IRR (95% CI) | reference | 2.07 (0.48–8.97) | 0.268 |
| log-rank test |  |  | 0.559 |
| MBI-LCBI | 0 (0%) | 0 (0%) |  |
| IR (95% CI) per 1,000 CVAD-days | 0.00 | 0.00 |  |
| IRR (95% CI) |  |  |  |
| log-rank test |  |  |  |
| Secondary BSI | 5 (3%) | 1 (2%) | 1.000^d^ |
| CRBSI | 3 (2%) | 0 (0%) | 0.567^d^ |
| IR (95% CI) per 1,000 CVAD-days | 1.35 (0.44–4.19) | 0.00 |  |
| IRR (95% CI) | reference | 0.00 (0.00–5.00) | 0.306 |
| log-rank test |  |  | 0.249 |
| *Note: frequencies and column percentages shown unless otherwise noted; percentages calculated with the number of non-missing values in the denominator; ^a^ row percentages shown; ^b^ median (25^th^–75^th^ percentiles) shown; ^c^ Wilcoxon rank-sum test; ^d^ Fisher’s exact test; ^e^ 14 patients had more than one common contaminant; ^f^ one patient was classified with a CLABSI and a MBI-LCBI; BSI = bloodstream infection; CFU = colony-forming unit; CI = confidence interval; CLABSI = central line-associated BSI; CRBSI = catheter-related BSI; CVAD = central venous access device; IR = incidence rate; IRR = incidence rate ratio; IVFE = intravenous fat emulsion; LCBI = laboratory confirmed bloodstream infection; MBI-LCBI = mucosal barrier injury LCBI.* | | | |
